# Preoperative computed tomography-guided localization pulmonary nodules: comparison between accura and soft anchored wires

**DOI:** 10.3389/fsurg.2025.1545503

**Published:** 2025-05-09

**Authors:** Feng Zhou, Jun Tao, Fei Shen, Xiang-Zhong Huang, Wen-Jia Huang, Fu-Lei Gao

**Affiliations:** ^1^ Department of Radiology, Jiangyin Hospital Affiliated to Nantong University, Jiangyin, China; ^2^ Department of Respiratory, Jiangyin Hospital Affiliated to Nantong University, Jiangyin, China; ^3^ Department of Thoracic Surgery, Jiangyin Hospital Affiliated to Nantong University, Jiangyin, China; ^4^ Department of Interventional Radiology, Jiangyin Hospital Affiliated to Nantong University, Jiangyin, China; ^5^ Department of Radiology, Xuzhou Central Hospital, Xuzhou, China

**Keywords:** computed tomography, localization, needle, pulmonary nodule, resection

## Abstract

**Background:**

Both Accura wire and soft anchored wire strategies can be employed for the preoperative computed tomography (CT)-guided localization of pulmonary nodules (PNs). The relative outcomes associated with these two distinct localization strategies, however, remain uncertain. This study thus sought to explore the relative safety and efficacy of preoperative CT-guided Accura wire and soft anchored wire localization for PNs.

**Methods:**

This was a retrospective study enrolling patients from two centers. Consecutive patients with PNs who underwent preoperative CT-guided Accura wire or soft anchored wire localization followed by video-assisted thoracic surgery (VATS) resection between January 2022 and December 2023 were enrolled in these analyses. The comparison was carried out between these two groups to evaluate the safety and efficacy.

**Results:**

Over the course of this study, 190 patients were enrolled and classified into the Accura wire (*n* = 100) and soft anchored wire (*n* = 90) groups. One PN was localized per patient, and the respective technical success rates for these two localization strategies were 98% (98/100) and 100% (*P* = 0.497). Dislodgement accounted for the two technical failures in the Accura wire group. Comparison of both groups revealed a comparable median localization procedural duration (9.0 vs. 9.0 min, *P* = 0.082), while the soft anchored wire group presented with visual analog scale scores significantly lower than those for the Accura wire group (3.0 ± 0.6 vs. 4.5 ± 0.6, *P* = 0.001). Significantly reduced pneumothorax (16.7% vs. 41%, *P* = 0.001) and pulmonary hemorrhage (23.3% vs. 41%, *P* = 0.01) rates were noted for the soft anchored wire relative to the Accura wire. VATS-guided limited resection was successfully performed for all patients.

**Conclusions:**

Both Accura wire and soft anchored wire strategies can facilitate accurate PN localization prior to VATS, although the latter strategy may be associated with a better safety profile relative to the former.

## Introduction

Pulmonary nodules (PNs) are non-transparent lesions in the lung parenchyma ≤3 cm in diameter not attributable to atelectasis, mediastinal lymphadenopathy, or pleural effusion (1–3). Video-assisted thoracic surgery (VATS) is the most accurate means of diagnosing PNs (4–6), with VATS-based limited wedge or segmental resection generally being sufficient for diagnosis. VATS limited resection can also be used to treat PNs in patients with tumors that are still minimally invasive. VATS limited resection technical success rates are generally improved via preoperative computed tomography (CT)-guided localization of the target PNs (7).

Many different materials have been used to assist in CT-guided PN localization (8), with the most widely used materials including different types of wire used for the localization of breast lesions such as Accura wire and hook wire (8). There are certain disadvantages to these worse, however. For one, their rigidity tends to cause patient discomfort, and it can also play an important role in the high rates of complications associated with localization procedures. In addition, there is no hook at the distal end of the Accura or hook wires, which can lead to their becoming dislodged as occurs in ∼6% of cases (8).

To address these issues, some research teams have explored the development of soft anchored wire for specific PN localization (9), which allows for the localization of target nodules using an approach similar to that for Accura or hook wire using improved materials. This approach uses an anchor consisting of four blunt claws that can attach the parenchyma of the lung around the target PN more securely. In addition, a soft, smooth suture is attached to this anchor in place of a hard steel wire, minimizing damage to the surrounding tissue and thereby potentially improving patient comfort following localization.

This study was developed with the goal of assessing the relative safety and efficacy of preoperative CT-guided Accura wire and soft anchored wire localization of PNs.

## Methods

### Study design

The Ethics Committees of Jiangyin Hospital affiliated with Nantong University and Xuzhou Central Hospital approved this two-center study and waived the need for written informed consent.

Consecutive patients with PNs who underwent preoperative CT-guided Accura wire or soft anchored wire localization from January 2022 and December 2023 followed by VATS resection were enrolled in this study. These patients were separated into two groups based on the type of wire utilized for localization. Patients eligible for inclusion were those (a) with PNs measuring ≤30 mm in size; (b) with PNs lacking a definitive pathological basis; and (c) patients with PNs deemed high risk according to the Lung-RADS assessment (2). Patients were excluded in cases where: (a) the distance from the PN to the pleura exceeded 4 cm; (b) the PNs were found to be <6 mm in diameter; (c) typical metastatic PNs were observed, or (d) patients exhibited severe comorbidities. Baseline, localization-related, and VATS-related data were compared between both groups.

### CT-guided localization

All procedures were performed by interventional radiologists having experience for more than 5 years in the field of conducting CT-guided interventional procedures. Localization was carried out under local anesthesia using a 16-slice CT instrument (Siemens, Berlin, Germany). After positioning patients appropriately according to the location of the target PN, the needle pathway was chosen according to the most direct path from the skin to the target nodule while avoiding the ribs, large blood vessels, and large blood vessels.

**Figure 1:**
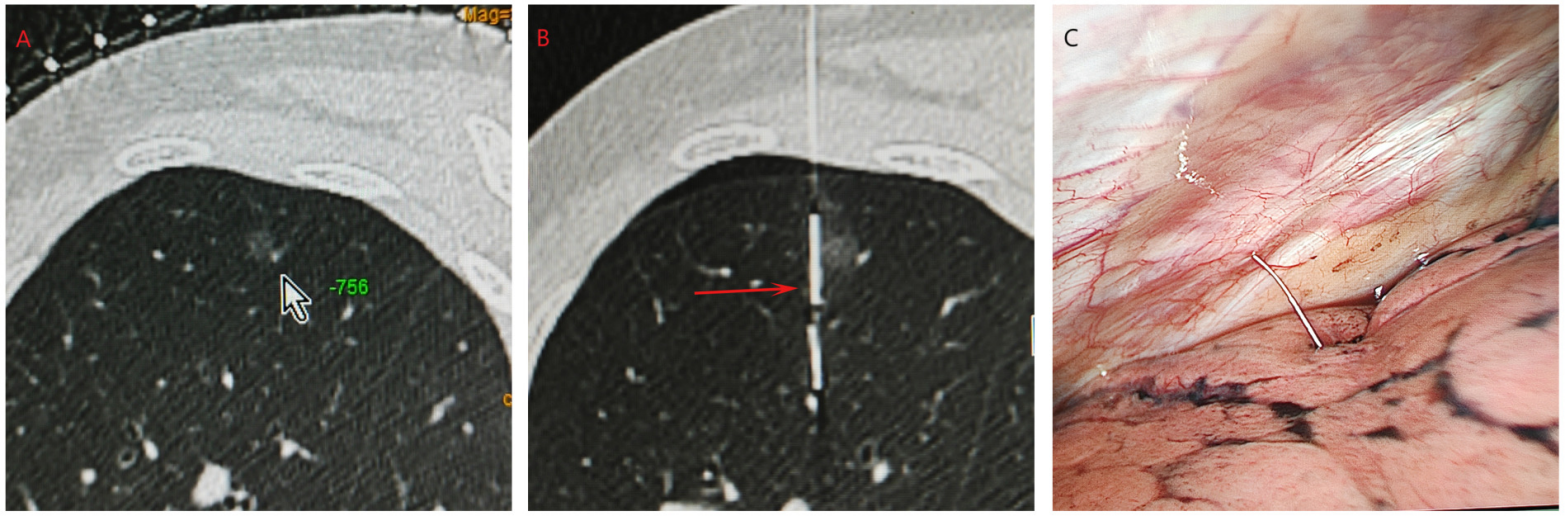
The procedures of CT-guided accura wire localization. **(a)** A GGN located at left upper lobe (arrow). **(b)** The Accura wire (red arrow) was placed near the GGN. **(c)** The Accura wire could be visualized during the VATS.

For Accura wire localization, a 21G Accura needle (Argon Medical Device, TX, USA) was utilized. This needle was used to puncture the lung parenchyma along the predetermined needle pathway, performing repeated CT scans to ensure the correct positioning of the needle tip. Following this, the tip of this needle was carefully advanced until it reached a point within 1 cm of the target nodule, whereupon the position of the tip was confirmed and the Accura wire was released [Figures 1a–c].

**Figure 2:**
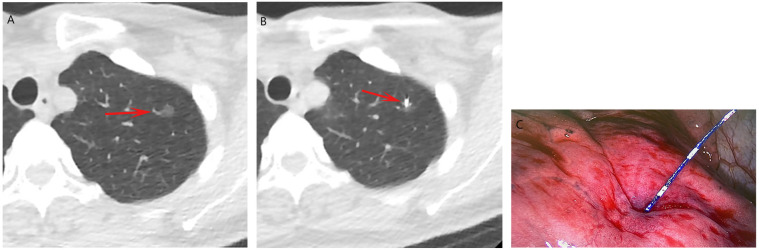
The procedures of CT-guided soft anchored wire localization. **(a)** A GGN located at left upper lobe (arrow). **(b)** The soft HW (arrow) was placed near the GGN. **(c)** The soft HW could be visualized during the VATS.

Soft anchored wire localization procedures were conducted using a 20G puncture needle (Senscure, Ningbo, China). Needle puncture procedures identical to those for the Accura wire group were utilized, and the anchor was released into the lung parenchyma proximal to the target lesion by removing a safety buckle and pressing the appropriate plunger. Upon withdrawing the needle, a three-colored suture remained in place within the needle tract. As this suture was longer than the distance from the needle tip to the pleura along the selected puncture path, the end of this suture was still visible on the exterior of the pleura [Figures 2a–c].

After localization was complete, an additional CT scan was conducted in an effort to observe any complications associated with the localization.

### VATS

VATS limited wedge or segmental resection procedures were generally conducted within 3 h of the completion of localization. During these procedures, the operators firstly identified the localization marker and used their fingers to palpate the inter-pulmonary localizer before resection from the utility incision. The operators successfully confirmed the location of the target PB according to the distance from this localizer to the nodule on CT examination. In cases where there was a margin of more than 2 inches between the target PN and the pleura, patients generally underwent segmental resection, whereas wedge resection was otherwise carried out. Resected nodules were brought to the Pathology department for detailed and rapid evaluation. When PNs were diagnosed as invasive lung cancer, patients underwent further lobectomy and lymphadenectomy. When PNs were diagnosed as minimally invasive lung cancer, sampling of the lymph node was subsequently conducted. All other PN types did not warrant any further procedures after limited resection.

### Assessment

CT-guided localization was deemed successful when all of the following were true: (a) operators were able to visualize the placed localizers while performing VATS procedures; (b) the occurrence of the localizer dislodgement was not observed, and (c) target PNs were confirmed to be located in the resected segment of the lung parenchyma. The duration of the CT-guided localization procedure was measured from the first CT scan to the final CT scan. Pain severity during this procedure was assessed while using the visual analog scale (VAS) with a scoring range of 0–10. The duration of the VATS procedure was recorded from the initial incision to the closure of the wound.

Rates of technical success for CT-guided localization procedures were the primary study endpoint, whereas secondary endpoints included localization procedure duration, localization-associated complications, VAS scores, VATS procedure duration, final diagnosis, and surgery type.

### Statistical analyses

All data were analyzed with SPSS 16.0 (SPSS, Inc., IL, USA). When normally distributed, continuous data were reported as mean (standard deviations) and compared using Student's *t*-tests, whereas they were otherwise reported as median (Q1; Q3) and compared using Mann–Whitney *U* tests. Categorical data were analyzed using *χ*^2^ or Fisher's exact tests. Risk factors related to pneumothorax incidence were identified through multivariate logistic regression analyses incorporating appropriate (*P* < 0.1) variable risk factors from univariate analyses. *P* < 0.05 was considered significant.

## Results

### Patients

In total, 190 patients were enrolled in this study and separated into the Accura wire (*n* = 100) and soft anchored wire (*n* = 90) groups. All patients underwent the localization of a single PN (Table 1). All measured baseline data other than the nodule-pleura distance were identical in both groups.

**Table 1:** Baseline characteristics between 2 groups.

Vaiables	Accura wire group	Soft anchored wire group	*P*
Patients number	100	90	
Age (y)	56.7 ± 12.0	55.8 ± 11.8	0.604
Gender			0.121
Male	59	43	
Female	41	47	
Smoking history	10	15	0.175
Malignant history	7	6	0.928
Diameter (mm)	10.3 ± 4.2	9.5 ± 2.0	0.068
Nodule-pleura distance (mm)	8.0 (Q1: 2.0; Q3: 17.4)	12.0 (Q1: 6.0; Q3: 20.0)	0.012
Nature of the nodules			0.053
Solid	22	28	
Mixed GGN	34	17	
Pure GGN	44	45	
Sides of the lung			0.213
Left	49	36	
Right	51	54	
Lobes			0.811
Upper	65	57	
Non-upper	35	33	

GGN, ground glass nodule.

### Localization outcomes

In the Accura wire and soft anchored wire groups, the respective technical success rates for localization procedures were 98% (98/100) and 100% (*P* = 0.497, Table 2). Technical failures arose in the Accura wire group as a consequence of dislodgement. Similar median localization procedure durations were evident in both groups (9.0 min vs. 9.0 min, *P* = 0.082), but VAS scores in the soft anchored wire group were significantly lower when compared with those for the Accura wire group (3.0 ± 0.6 vs. 4.5 ± 0.6, *P* = 0.001).

**Table 2:** Comparison of localization-related data.

Variables	Accura wire group	Soft anchored wire group	*P*
Successful localization rate	98% (98/100)	100% (90/90)	0.497
Dislodgement	2 (2%)	0 (0%)	0.497
Duration of localization (min)	9.0 (Q1: 7.0; Q3: 11.0)	9.0 (Q1: 8.0; Q3: 11.8)	0.082
VAS	4.5 ± 0.6	3.0 ± 0.6	0.001
Complications			
Pneumothorax	41% (41/100)	16.7% (15/90)	0.001
Pulmonary hemorrhage	41% (41/100)	23.3% (21/90)	0.01

VAS, visual analog scale.

Pneumothorax rates in the Accura wire group were found to be significantly higher when compared with those in the soft anchored wire group (41% vs. 16.7%, *P* = 0.001), but these pneumothorax episodes did not affect the VATS procedure. Following univariate and multivariate analysis analyses (Table 3), upper lobe location (*P* = 0.03) and soft anchored wire utilization (*P* = 0.002) were identified as protective factors related to pneumothorax risk.

**Table 3:** Predictors of pneumothorax.

Variables	Univariate analysis			Multivariate analysis		
	Hazard ratio	95% CI	*P* value	Hazard ratio	95% CI	*P* value
Age	0.990	0.965–1.017	0.472			
Gender						
Male	1					
Female	0.964	0.516–1.802	0.909			
Smoking history	0.499	0.179–1.392	0.184			
Tumor history	1.074	0.437–2.637	0.876			
Diameter	1.115	1.019–1.220	0.018	1.086	0.989–1.193	0.083
Nodule-pleura distance	0.989	0.960–1.019	0.470			
Nature of the nodules						
Solid	1					
Pure GGN	0.813	0.356–1.857	0.623			
Mixed GGN	0.584	0.275–1.238	0.161			
Lung sides						
Right	1					
Left	1.393	0.745–2.606	0.300			
Lung lobes						
Non-upper	1			1		
Upper	0.545	0.288–1.032	0.063	0.471	0.238–0.931	0.03
Duration of localization	1.021	0.967–1.078	0.454			
Localization material						
Accura wire	1			1		
Soft anchored wire	0.288	0.145–0.570	0.001	0.323	0.158–0.661	0.002

GGN, ground glass nodule.

Pulmonary hemorrhage was significantly more common among patients in the Accura wire group as compared to the soft anchored wire group (41% vs. 16.7%, *P* = 0.001), but these hemorrhage incidents had no effect on the VATS procedure. Univariate and multivariate analyses failed to reveal any specific factors linked to the risk of pulmonary hemorrhage (Table 4).

**Table 4:** Predictors of lung hemorrhage.

Variables	Univariate analysis			Multivariate analysis		
	Hazard ratio	95% CI	*P* value	Hazard ratio	95% CI	*P* value
Age	1.008	0.983–1.035	0.535			
Gender						
Male	1			1		
Female	0.582	0.316–1.073	0.083	0.759	0.391–1.477	0.417
Smoking history	0.850	0.350–2.066	0.720			
Tumor history	0.332	0.109–1.011	0.052	0.511	0.159–1.642	0.260
Diameter	1.068	0.980–1.164	0.133			
Nodule-pleura distance	1.023	0.995–1.052	0.109			
Nature of the nodules						
Solid	1			1		
Pure GGN	3.989	1.560–10.169	0.004	3.234	0.939–8.446	0.077
Mixed GGN	2.947	1.233–7.044	0.015	2.788	0.851–6.751	0.093
Lung sides						
Right	1					
Left	0.961	0.521–1.771	0.898			
Lung lobes						
Non-upper	1					
Upper	1.055	0.561–1.985	0.868			
Duration of localization	1.021	0.968–1.078	0.444			
Localization material						
Accura wire	1			1		
Soft anchored wire	0.438	0.233–0.823	0.001	0.564	0.284–1.122	0.103

GGN, ground glass nodule.

### VATS outcomes

VATS-guided limited resection was successfully performed for all patients in this study. While CT-guided Accura wire localization was not a technical success for two patients, both were able to undergo VATS-guided resection successfully as the operators were able to intraoperatively identify the site of bleeding on the visceral pleura attributable to the puncture needle. Conversion to thoracotomy did not occur in any case. VATS procedure types are detailed in Table 5. In total, 21 and 31 patients in the Accura wire and soft anchored wire groups, respectively, underwent subsequent lobectomy owing to the diagnosis of invasive adenocarcinoma. Table 5 presents details regarding the pathological diagnoses for all PNs.

**Table 5:** Comparison of VATS related data.

Variables	Accura wire group	Soft anchored wire group	*P*
Technical success of limited resection	100% (100/100)	100% (90/90)	
Duration of VATS (min)	55 (Q1:40.0; Q3: 75.0)	82.5 (Q1: 60.0; Q3: 116.3)	0.001
Surgical types			0.139
Wedge resection	66	53	
Segmental resection	13	6	
Wedge resection + lobectomy	10	16	
Segmental resection + lobectomy	11	15	
Final diagnoses			0.034
Invasive adenocarcinoma	21	31	
Mini-invasive adenocarcinoma	40	22	
Adenocarcinoma *in situ*	7	14	
Metastasis	1	0	
Precancerous lesion	6	7	
Benign	25	16	

VATS, video-assisted thoracoscopic surgery.

## Discussion

Wire-like materials are the most frequently used approach to localizing PNs as they allow for straightforward localization procedures. Moreover, these localization materials allow for localization at both the PN and the pleura. Liquid localization materials can also be placed with ease, but only offer the advantage of pleural localization without any corresponding localization proximal to the target nodule. Microcoils can provide localization information for both of these sites, but their placement during the localization procedure is more technically difficult.

This study included comparisons of clinical efficacy and safety between the CT-guided Accura wire and soft anchored wire-based localization of PNs. Both groups exhibited similar technical success rates, demonstrating that both strategies can reliably localize these nodules. Zhou et al. (10) also demonstrated similar rates of success with respect to the localization of PNs using hook-wire and soft anchored wire approaches (95.2% vs. 99.1%, *P* = 0.117), although they observed a significantly higher rate of dislodgement for the former group relative to the latter (4.8% vs. 0.0%, *P* = 0.029). While the dislodgement rates in the present study did not differ significantly between the Accura and soft anchored wire groups (*P* = 0.497), the soft anchored wire was not associated with any instances of dislodgement. This is attributable to the four blunted claws used in the design of this soft anchored wire.

Localization-associated VAS scores and complication rates were both lower in the soft anchored wire group relative to the Accura wide group, primarily owing to the rigidity of the Accura wire. The soft anchored wire group made use of soft sutures in place of hard steel wire, improving patient comfort and lowering the associated rates of complications.

In addition to soft anchored wire utilization, PNs located in the upper lobe were established as a factor associated with a reduction in the risk of localization-related pneumothorax (*P* = 0.03). Han et al. (11) additionally determined that upper lobe location was associated with the risk of localization-related pneumothorax. This may be because the lower lungs play a more important role in respiratory motion relative to the upper lungs (12). However, factors associated with the enhancement or suppression of pulmonary hemorrhage risk were not identified in this study, possibly due to the limitation of the sample size.

The diameter of the puncture needle may also be associated with the complication rate. However, the soft anchored wire was corresponded to the 20G puncture needle, while the Accura wire was corresponded to the 21G puncture needle. Therefore, the diameter of the puncture needle may not be associated with the complications in this study.

While the localization process was a technical failure for two of the patients in the Accura wire group, VATS-guided limited resection was successfully performed in both cases. This is consistent with the fact that in addition to resection successfully being performed based on the localization marker when localization is successful, the intraoperative identification of the puncture site can still enable VATS resection in some instances of technical failure (13), although this may result in the prolongation of the VATS procedure. The soft anchored wire group in this study exhibited a significantly longer mean VATS duration relative to the Accura wire group (82.5 min vs. 55.0 min, *P* = 0.001), possibly because the former group enrolled more patients diagnosed with invasive adenocarcinomas who needed to undergo lobectomy.

There are certain limitations to this study. For one, this was a retrospective analysis and it is thus susceptible to selection bias, underscoring the need for additional prospective randomized controlled trials. Secondly, there was an imbalance in nodule-pleura distance when comparing the two groups, potentially compounding the risk of bias. Third, while this study enrolled patients from two centers, there may have been differences in the CT-guided localization and VATS resection experience of the operators that treated these patients, providing another source of possible bias.

## Conclusion

In summary, these results suggest that both Accura wire and soft anchored wire can aid in accurate PN localization before VATS. Of these two strategies, however, soft anchored wire use for localization can potentially offer an improved safety profile.

## Data Availability

The original contributions presented in the study are included in the article/Supplementary Material, further inquiries can be directed to the corresponding author/s.
